# Degradation Dynamics of Glyphosate in Different Types of Citrus Orchard Soils in China

**DOI:** 10.3390/molecules20011161

**Published:** 2015-01-12

**Authors:** Changpeng Zhang, Xiuqing Hu, Jinyan Luo, Zhiyi Wu, Li Wang, Bin Li, Yanli Wang, Guochang Sun

**Affiliations:** 1 MOA Key Lab for Pesticide Residue Detection, Institute of Quality and Standard for Agro-products, Zhejiang Academy of Agricultural Sciences, Hangzhou 310021, China; E-Mails: cpzhang1215@126.com (C.Z.); huxq@mail.zaas.ac.cn (X.H.); 2 Shanghai Extension and Service Center of Agriculture Technical, Shanghai 201103, China; E-Mail: toyanzi@126.com; 3 Zhejiang Entry-Exit Inspection and Quarantine Bureau, Hangzhou 310012, China; E-Mail: mkunwu@163.com; 4 State Key Laboratory of Rice Biology, Institute of Biotechnology, Zhejiang University, Hangzhou 310058, China; E-Mail: wangli028820@163.com; 5 State Key Laboratory Breeding Base for Zhejiang Sustainable Plant Pest and Disease Control, Key Laboratory of Detection for Pesticide Residues, Ministry of Agriculture, Zhejiang Academy of Agricultural Sciences, Hangzhou 310021, China; E-Mail: ylwang88@aliyun.com

**Keywords:** degradation dynamics, residue, glyphosate, soil properties, HPLC

## Abstract

Glyphosate formulations that are used as a broad-spectrum systemic herbicide have been widely applied in agriculture, causing increasing concerns about residues in soils. In this study, the degradation dynamics of glyphosate in different types of citrus orchard soils in China were evaluated under field conditions. Glyphosate soluble powder and aqueous solution were applied at 3000 and 5040 g active ingredient/hm^2^, respectively, in citrus orchard soils, and periodically drawn soil samples were analyzed by high performance liquid chromatography. The results showed that the amount of glyphosate and its degradation product aminomethylphosphonic acid (AMPA) in soils was reduced with the increase of time after application of glyphosate formulations. Indeed, the amount of glyphosate in red soil from Hunan and Zhejiang Province, and clay soil from Guangxi Province varied from 0.13 to 0.91 µg/g at 42 days after application of aqueous solution. Furthermore, the amount of glyphosate in medium loam from Zhejiang and Guangdong Province, and brown loam from Guizhou Province varied from less than 0.10 to 0.14 µg/g, while the amount of AMPA varied from less than 0.10 to 0.99 µg/g at 42 days after application of soluble powder. Overall, these findings demonstrated that the degradation dynamics of glyphosate aqueous solution and soluble powder as well as AMPA depend on the physicochemical properties of the applied soils, in particular soil pH, which should be carefully considered in the application of glyphosate herbicide.

## 1. Introduction

Glyphosate (*N*-(phosphonomethyl) glycine), which contains several dissociable hydrogens, is the active ingredient in herbicide formulations containing it. Indeed, glyphosate or glyphosate formulations such as Roundup are able to kill weeds without killing their crops, especially annual broadleaf weeds and grasses known to compete with commercial crops grown around the globe by interfering with the synthesis of the aromatic amino acids phenylalanine, tyrosine and tryptophan [1,2,3,4,5,6,7,8]. Nowadays, glyphosate formulations that are used as a broad-spectrum systemic herbicide have been widely applied in agronomic crops and orchards. Furthermore, glyphosate formulations are currently marketed in the worldwide by many agrochemical companies, such as Bayer, Dow Agro Sciences, and Monsanto in different solution strengths and with various adjuvants. In addition, it has been reported to be the world's largest selling herbicide in 2013, and Chinese manufacturers are the world's largest producers of glyphosate and its precursors [1,2,3,4,5,6,7,8].

The United States Environmental Protection Agency and the European Union consider that there is no potential for the herbicide glyphosate to pose a health risk to humans. Furthermore, early epidemiological studies did not find associations between long term low level exposure to glyphosate and any disease [4,5,9], but with the heavy use of fungicides, insecticides and herbicides such as the glyphosate formulation Roundup in agriculture, residues in soils are a growing problem [10,11,12,13,14,15,16,17,18]. Indeed, a field test showed that lettuce, carrots, and barley contained glyphosate residues up to one year after the soil was treated with 4.15 kg of glyphosate per hectare [6,7]. Glyphosate is readily degraded by soil microbes to carbon dioxide and aminomethylphosphonic acid (AMPA) that adsorbs strongly to soil and is not expected to move vertically below the top six inch soil layer. Therefore, studying the behavior of glyphosate in the soil environment has great significance for its valid application and environmental safety evaluation.

To our knowledge, little information is available about the dissipation of glyphosate from different formulations under field conditions. The aim of this research was to examine the degradation dynamics of glyphosate aqueous solution, soluble powder and its metabolite AMPA, in different type of soils found in citrus orchards in China. 2. Results and Discussion

## 2. Results and Discussion

### 2.1. Recovery Percentage of Glyphosate and Its Metabolite AMPA

In this study, the amount of glyphosate and AMPA in different types of orchard soils was determined by HPLC, which has been widely used for the measurement of residues [19,20,21,22,23,24,25,26]. The minimum detectable amount of glyphosate and AMPA residues was 1 × 10^−11^ g in the HPLC analysis. Furthermore, the minimum concentration of glyphosate and AMPA detection was 0.10 mg/kg, which has been regarded as a criterion for the safety of humans and animals. The relative retention times of glyphosate and AMPA were 18.818 and 11.781 min, respectively. The recovery percentage of glyphosate varied from 95.60% to 98.82%, while the recovery percentage of AMPA varied from 97.60% to 99.60% after applying 99% glyphosate standard sample to an orchard soil from Zhejiang Province (Table 1). Since the recovery percentage was more than 80.00%, the residue data from the soil samples have not been corrected for the recoveries. This result justified that the HPLC method could to be used for the detection of glyphosate and AMPA residue in Chinese soils.

**Table 1:** Recovery percentage of glyphosate and its metabolite AMPA from soils in Zhejiang Province.

Concentration of Glyphosate (mg/kg)	Recovery Percentage (%)	
	Glyphosate	AMPA
0.05	95.60 ± 3.23	97.60 ± 4.83
0.50	96.40 ± 3.67	98.20 ± 4.72
5.00	98.82 ± 4.16	99.60 ± 4.91

Mean of five replicates.

### 2.2. Standard Curve of Glyphosate and Its Metabolite AMPA

In order to quantify the amount of glyphosate and AMPA residue in different types of orchard soils, glyphosate and AMPA solutions at different concentrations were prepared for HPLC analysis by dissolving a 99% glyphosate standard sample into ddH_2_O and extracts of Zhejiang soil, respectively. Furthermore, a standard curve of glyphosate and AMPA was drawn based on the peak area-concentration determined of HPLC by the least squares method.

**Figure 1:**
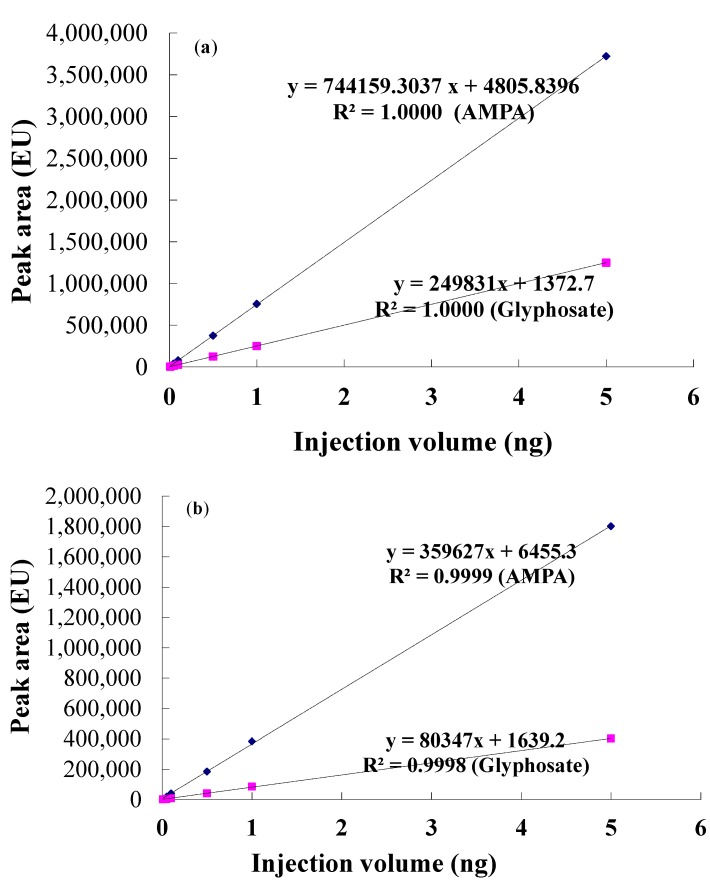
Standard curves of glyphosate and AMPA (**a**) ddH_2_O and (**b**) soil samples.

In this study, the standard curve of glyphosate and AMPA in ddH_2_O was represented as y = 249,831x + 1372.7 (R² = 1.0000) and y = 744,159.3037x + 4805.8396 (R² = 1.0000), respectively, while glyphosate and AMPA in soil was represented by the equation y = 80,347x + 1639.2 (R² = 0.9998) and y = 359,627x + 6455.3 (R² = 0.9999), respectively (Figure 1).

### 2.3. Degradation Dynamics of Glyphosate Soluble Powder

**Figure 2:**
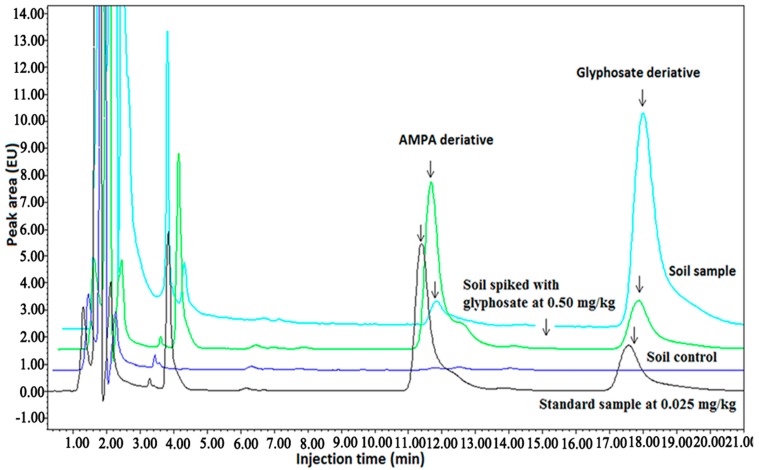
Chromatogram of glyphosate and AMPA standard and soil samples in 2008 field experiments.

Result from this study indicated that no glyphosate was detected in the control soil samples, where no glyphosate formulation was sprayed. This result suggests that the level of glyphosate in the natural orchards of different sites in China was lower than 0.10 mg/kg, which has been regarded as the safety criterion for humans and animals (Figure 2). In general, after spraying glyphosate, the content of glyphosate in the soils decreased as time increased regardless of the type of soils. In particular, the level of glyphosate in medium loam from Guangdong Province was lower than 0.10 mg/kg at 42 days after the spraying of glyphosate (Table 2).

There was no extreme rain or storms during the spraying period, indicating the degradation is mainly due to the soil itself. This result indicated that the glyphosate soluble powder could be well degraded in the three kinds of soils although there was a slight difference in the degradation dynamics of the glyphosate soluble powder between the three kinds of soils. Indeed, the results from this study indicated that the amount of glyphosate residue is less than 0.10 mg/kg in medium loam from Guangdong, and nearly 0.10 mg/kg in medium loam from Zhejiang Province and in brown loam from Guizhou Province at 42 days after the spraying of this herbicide. In addition, the half-lives of glyphosate soluble powder in soils from Zhejiang Province, Guangdong Province and Guizhou Province are 12.6 days, 11.7 days and 10.0 days, respectively (Table 2).

**Table 2:** Degradation dynamics of glyphosate soluble powder in different type of soils.

Days after Spraying	Residue of Glyphosate (mg/kg)		
	Medium Loam (Zhejiang)	Medium Loam (Guangdong)	Brown Loam (Guizhou)
1	1.32 ± 0.11	1.25 ± 0.10	2.07 ± 0.16
3	0.92 ± 0.09	0.60 ± 0.03	2.01 ± 0.16
7	0.56 ± 0.18	0.40 ± 0.02	1.92 ± 0.18
14	0.38 ± 0.10	0.32 ± 0.01	0.75 ± 0.11
21	0.18 ± 0.05	0.26 ± 0.01	0.32 ± 0.03
28	0.18 ± 0.03	0.19 ± 0.02	0.30 ± 0.03
35	0.15 ± 0.01	0.10 ± 0.01	0.25 ± 0.04
42	0.13 ± 0.01	<0.10	0.14 ± 0.03
T_1/2_ (day)	12.6	11.7	10.0
Equation	C_t_ = 0.9501e^−0.^^0550t^ (R^2^ = 0.8926)	C_t_ = 0.8452e^−0.^^0594t^ (R^2^ = 0.8996)	C_t_ = 2.2562e^−0.^^0690t^ (R^2^ = 0.9446)

In agreement with the data in this study, previous studies indicated that the persistence of pesticides, fungicides and herbicides in soil was affected by a number of factors such as climate conditions and soil type [27,28,29,30]. Furthermore, half-lives varied from as little as 3 days at a soil site in Texas (USA) to 141 days at a soil site in Iowa (USA) [1,2,3,4,5,6,7,8]. In addition, a 2009 study using a Roundup formulation concluded that absorption into plants delays subsequent soil-degradation and can increase the persistence of glyphosate in soil from two to six times. The half-life of glyphosate in soil ranges between 2 and 197 days, while a typical field half-life of 47 days has been suggested [6,7]. Obviously, these results indicate that glyphosate adsorption in soil, and later release from soil depends on the kind of soil. Interestingly, result from this study clearly indicated that the half-lives of glyphosate soluble powder in different types of citrus orchard soils in China are less than the typical field half-life.

### 2.4. Degradation Dynamics of AMPA

Result from this study indicated that no AMPA was detected in the control soil samples where no glyphosate formulation was sprayed, suggesting that the level of AMPA in the tested orchards of different sites in China was lower than 0.10 mg/kg. In general, after spraying glyphosate, the amount of AMPA residue in the soils decreased with the increase of time regardless of the type of soils. In particular, the level of AMPA was lower than 0.10 mg/kg in the soil sample from Guangdong Province at one day after the spraying of glyphosate, suggesting that the glyphosate soluble powder and its metabolite AMPA were well degraded in this kind of soil (Table 3).

**Table 3:** Degradation dynamics of glyphosate soluble powder AMPA in different type of soils.

Days after Spraying	Residue of Glyphosate AMPA (mg/kg)		
	Medium Loam (Zhejiang)	Medium Loam (Guangdong)	Brown Loam (Guizhou)
1	2.99 ± 0.16	<0.10	3.21 ± 0.34
3	1.85 ± 0.08	<0.10	2.25 ± 0.21
7	1.72 ± 0.11	<0.10	1.34 ± 0.16
14	1.52 ± 0.03	<0.10	1.08 ±0.18
21	1.43 ± 0.04	<0.10	0.97 ± 0.25
28	1.41 ± 0.13	<0.10	0.91 ± 0.16
35	1.26 ± 0.09	<0.10	0.62 ± 0.09
42	0.99 ± 0.01	<0.10	0.33 ± 0.03
T_1/2_ (day)	36.9	/	10.0
Equation	C_t_ = 2.2347e^−0.^^0188t^ (R^2^ = 0.7811)	/	C_t_ = 2.5019e^−0.^^0444t^ (R^2^ = 0.9041)

There was no extreme rain or storms during the spraying period, indicating that the residue is mainly due to the soil itself. In general, this result found that there was a marked difference in the degradation dynamics of the AMPA between the three kinds of soils. Indeed, the amount of AMPA residue in medium loam from Zhejiang Province is 0.99 mg/kg, while the residue of AMPA in brown loam from Guizhou Province is 0.33 mg/kg at 42 days after the spraying of glyphosate. Furthermore, the half-lives of AMPA in soils from Zhejiang Province and Guizhou Province are 36.9 days and 10 days, respectively. However, at one day after the spraying of glyphosate, the level of AMPA was lower than 0.10 mg/kg in medium loam from Guangdong Province (Table 3).

Interestingly, the difference in degradation dynamics of AMPA was not only found between the medium loam and brown loam, but also was found between the medium loam from Zhejiang Province and the medium loam from Guangdong Province (Table 3). This difference in degradation dynamics between the same soil types may be due to their physicochemical properties. Indeed, there was a difference in pH, cation exchange capacity, the organic matter and soil texture between the two medium loams, justifying the difference in degradation of AMPA.

### 2.5. Degradation Dynamics of Aqueous Solution

**Figure 3:**
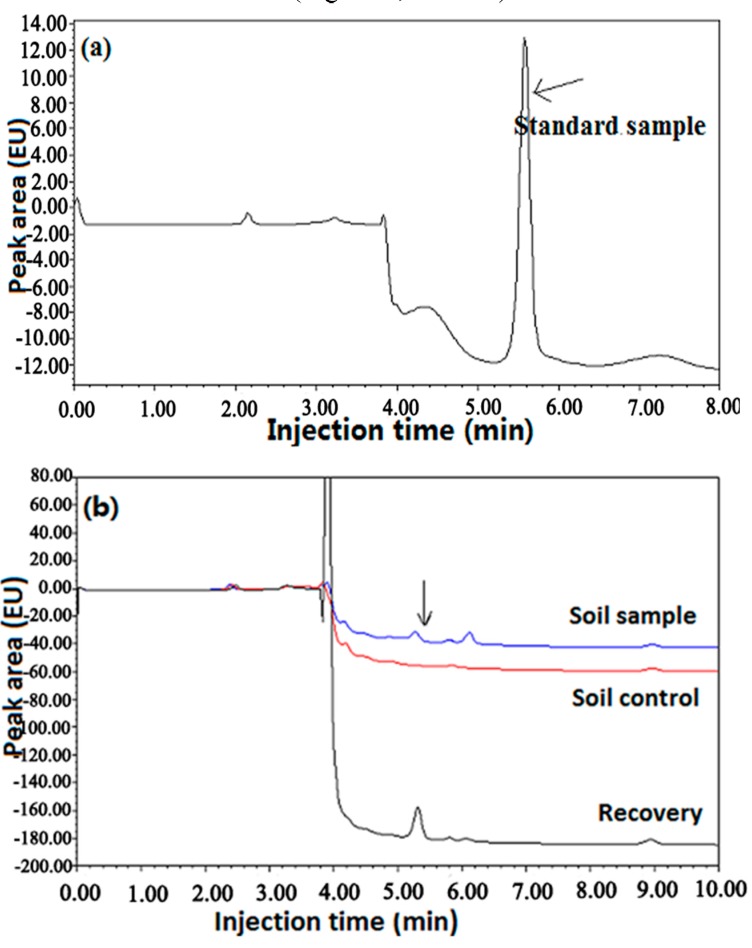
Chromatogram of glyphosate (**a**) ddH_2_O and (**b**) soil samples in 2011 field experiments.

Result from this study indicated that no glyphosate was detected in the control soil samples where no glyphosate formulation was sprayed. This result suggests that the level of glyphosate in the natural orchards of different sites in China was lower than 0.10 mg/kg. In general, after spraying the glyphosate formulation, the content of glyphosate in the soils decreased with the increase of time regardless of the type of soils. Indeed, the residue varied from 9.10 to 0.13 mg/kg in red soil from Zhejiang Province, from 6.33 to 0.35 mg/kg in clay from Guanxi Province, and from 6.22 to 0.91 mg/kg in red soil from Hunan Province (Figure 3; Table 4).

The amount of glyphosate residue was higher than 0.10 mg/kg in the three kinds of soils, indicating that it was difficult for the glyphosate aqueous solution to be completely degraded in these soils. However, this result indicated that there was a marked difference in the degradation dynamics of the glyphosate aqueous solution between the three kinds of soils. Indeed, the amount of residue in the clay from Guanxi Province and red soil from Hunan Province was higher than that in red soil from Zhejiang Province, which was close to 0.10 mg/kg. Interestingly, a difference in degradation dynamics was also found between the red soil from Zhejiang Province and the red soil from Hunan Province (Table 4), which may be due to the different characteristics of soils. Indeed, there was a difference in pH, cation exchange capacity, the organic matter and soil texture between the two red soils, justifying their differences in degradation of glyphosate.

**Table 4:** Degradation dynamics of glyphosate aqueous solution in different type of soils.

Days after Spraying	Residue of Glyphosate (µg/g)		
	Red Soil (Zhejiang)	Clay (Guangxi)	Red Soil (Hunan)
1	9.10 ± 0.16	6.33 ± 0.17	6.22 ± 0.17
3	8.59 ± 0.09	5.30 ±0.23	5.43 ± 0.11
7	1.89 ± 0.09	4.91 ± 0.11	4.79 ± 0.05
14	1.09 ± 0.05	3.24 ± 0.04	2.06 ± 0.10
21	0.84 ± 0.03	2.50 ± 0.21	1.64 ± 0.25
28	0.77 ± 0.03	1.95 ± 0.18	1.41 ± 0.18
35	0.31 ± 0.02	1.39 ± 0.14	1.04 ± 0.09
42	0.13 ± 0.01	0.35 ± 0.06	0.91 ± 0.06
T_1/2_ (day)	7.5	11.8	14.2
Equation	C_t_ = 6.9105e^−0.^^0929t^ (R^2^= 0.9140)	C_t_ = 7.3944e^−0.^^0587t^ (R^2^= 0.8968)	C_t_ = 5.7277e^−0.^^0488t^ (R^2^= 0.9630)

There was no extreme rain or storms during the spraying period, indicating the residue is mainly due to the itself. This result revealed that the degradation dynamics of glyphosate aqueous solution was affected by the soil type. Indeed, the half-lives of glyphosate aqueous solution in soils from Zhejiang Province, Guangxi Province and Hunan Province are 7.5, 11.8 and 14.2 days, respectively (Table 4). In addition, compared to the glyphosate soluble powder, this amount of glyphosate aqueous solution residue in the three soils was very high at 3 days after the spraying of glyphosate formulation regardless of the soil type. This may be partially due to the differences in the original amount of glyphosate in the soil. Indeed, aqueous solution was sprayed at 5040 g active ingredient/hm^2^, while the soluble powder was sprayed at 3000 g active ingredient/hm^2^.

Previous studies indicated that substantial mortality occurred only at concentrations of glyphosate exceeding the expected environmental concentrations [1,4,5,9]. The most common formulation in agriculture is 360 g active ingredient per litre, either alone or with added cationic surfactants. For 360 g formulations, European regulations allow applications of up to 12 L (4320 g active ingredient) per hectare for control of perennial weeds such as *Cynodon dactylon*. More commonly, rates of 3 L (1080 g active ingredient) per hectare are practiced for control of annual weeds between crops. In this study, the high residue in different type of soils may be mainly attributed to the large amount (5040 g active ingredient/hm^2^) of application.

The difference in the amount between glyphosate soluble powder and aqueous solution residue may be also due to the other ingredients other than glyphosate in the glyphosate formulation. Indeed, glyphosate that as an acidic molecule that is formulated as a salt for packaging and handling, using isopropylamine, diammonium, monoammonium, or potassium species as the counterion. Furthermore, in addition to glyphosate salts, commercial formulations of glyphosate contain additives such as surfactants which vary in nature and concentration. Therefore, the type of glyphosate formulation should be considered in any study of glyphosate degradation, which will be helpful to compare data from different investigations.

In agreement with the results from this study, a number of previous ones [1,6,7,9] have indicated that degradation of glyphosate in soil environments was dependent on the soil properties. For example, Ghafoor *et al*., reported the influence of various soil physical, chemical and microbiological characteristics on the persistence of glyphosate in soil [31]. Furthermore, investigations have shown that less than 5% of added glyphosate was mineralized at 15 °C after 3 months in some soils, while in other soils more than 40% was mineralized under the same conditions, indicating that the rate of degradation is very soil dependent [32]. In addition, differences in the half-life of glyphosate have been widely reported, emphasizing the great variability among soils.

Considering the complexity of glyphosate degradation, it is also very necessary to develop more sophisticated models to describe the degradation of glyphosate in soil. Interestingly, a simple general model has recently been proposed for measurement of microbial degradation of pesticides in soil [33]. However, in this study, the half-life times of glyphosate have been successfully estimated by a first-order rate expression. In addition, this difference in characteristics of soils suggested that the sites were representative of different type of orchard soils in China, which will give an overall evaluation for the degradation dynamics of glyphosate soluble powder and aqueous solution in soil environments.

Previous studies have revealed that the main glyphosate degradation pathway in soils is due to various microorganisms, in particular bacteria, which, together with soil factors, affect the degradation outcome and rate of degradation [32,33]. Furthermore, some investigations have indicated that glyphosate degradation is correlated with the general microbial activity measured by the respiration rate or the total number of culturable microorganisms. However, it has been demonstrated that the degradation rate of glyphosate can exhibit great variation for different microorganisms. Indeed, some microbes, in particular soil bacteria such as *Arthrobacter atrocyaneus*, *Enterobacter cloacae*, *Bacillus cereus*, *Pseudomonas aeruginosa* and *Pseudomonas* sp., have been reported to be able to degrade glyphosate effectively [32,34,35,36].

The survival of degrading bacteria in soil depends on the soil type and properties, justifying the importance of soil properties in the degradation dynamics of glyphosate. In agreement with the result of Ghafoor *et al.* [31], this study revealed that glyphosate degradation was significantly positively correlated to soil pH. Indeed, the half-life times of glyphosate in neutral and alkali soil are less than that in acid soil, which may be attributed to the differences in the soil microbial community. As we know, bacteria are able to grow well under neutral and alkali conditions, therefore this result highlights the importance of soil bacteria in glyphosate degradation in soils.

## 3. Experimental Section

### 3.1. Glyphosate Formulations

Two kinds of commercial formulations of glyphosate were used in this study, which was purchased from a local pesticides shop. One was 50% glyphosate soluble powder, while the other was 18% glyphosate aqueous solution. The two commercial formulations were produced by Beijing Century Aoke Biotechnology Co. Ltd, Beijing, China. The degradation dynamics of glyphosate were evaluated by measuring the amount of their residues in different types of citrus orchard soils collected in China. A standard sample of glyphosate with 99.0% purity was purchased from Beijing Century Aoke Biotechnology Co. Ltd. All reagents for HPLC are analysis or chromatographic grade unless specifically stated.

### 3.2. Experimental Design of Soluble Powder Degradation

Field experiment I was performed from September to November 2008 at a citrus orchard located in Jiande City (Zhejiang Province, China), Guangzhou City (Guangdong Province, China) and Guiyang City (Guizhou Province, China), respectively. The fields were partitioned into two blocks for two trials. Each block was further partitioned into three subplots (5 m × 6 m separated by earth embankments). Citrus orchards soils were sprayed with 50% glyphosate soluble powder at 3000 g active ingredient/ha using a manual sprayer (Jacto HD400, Singapore). The control soil was sprayed with the same volume of distilled water. One kilogram of soil from 0–10 cm depth was collected from at least five different sites at 1 day, 3 days, 7 days, 14 days, 21 days, 28 days, 35 days, and 42 days after the spraying of herbicide. The soils samples were stored in −20 °C. Each plot had three replicates.

Measurement of soil characteristics indicated that there were marked differences in the type, pH, cation exchange capacity and organic matter of the soils from different orchards which were selected as the sites to examine the degradation dynamics of glyphosate in the Chinese soil environment. Indeed, the types of soils from Jiande, Guanzhou and Guizhou were medium, medium and brown loam, respectively, while the pH of soil from Jiande, Guangzhou and Guizhou was 5.61, 7.30 and 4.26, respectively.

Furthermore, the organic matter from soils of Jiande, Guanzhou and Guizhou was 1.91%, 2.65% and 4.69%, respectively, while the cation exchange capacity was 6.81, 23.21 and 10.10 cmol/kg, respectively. In addition, there was a difference in soil texture between the above three samples, while the sand content was 40.22%, 47.60% and 14.80%, respectively, the silt content was 44.50%, 14.20%, and 67.10%, respectively, and the clay content was 15.30%, 38.20% and 18.10%, respectively (Table 5).

**Table 5:** Characteristics of orchard soils that were sprayed with glyphosate soluble powder.

Soil Type	Site	pH	CEC (cmol/kg)	Organic Matter (g/kg)	Soil Texture		
					Sand (%)	Silt (%)	Clay (%)
Medium loam	Jiande, Zhejiang	5.61	6.81	19.1	40.22	44.50	15.30
Medium loam	Guangzhou, Guangdong	7.30	23.21	26.5	47.60	14.20	38.20
Brown loam	Guiyang, Guizhou	4.26	10.10	46.9	14.80	67.10	18.10

CEC: Cation exchange capacity.

### 3.3. Experimental Design of Aqueous Solution Degradation

Field experiment II was carried out from Octorber to December 2011 at citrus orchards located in Hangzhou City (Zhejiang Province, China), Nanning City (Guangxi Province, China) and Changsha City (Hunan Province, China), respectively. The field was partitioned into two blocks for two trials. Each block was further partitioned into three subplots (5 m × 6 m separated by earth embankments). Each subplot was separated by protected lines. Citrus orchard soils were sprayed with 18% glyphosate aqueous solution at 5040 g active ingredient/hm^2^ using a manual sprayer (Jacto HD400). The control soil was sprayed with the same volume of distilled water. Two kilograms of soil from 0–10 cm depth was randomly collected from at least five different sites (direction and layer) at 1 day, 3 days, 7 days, 14 days, 21 days, 28 days, 35 days, and 42 days after the spraying. The soil samples were sealed in plastic bags marked with numbers and stored at −20 °C. Each plot had three replicates.

There was a marked difference in the characteristics of the soils that were sprayed with glyphosate aqueous solution in the different orchards. Indeed, the types of soils from Hangzhou, Nanning and Changsha were red soil, clay and red soil, respectively, while the pH of soils from Hangzhou, Nanning and Changsha was 6.34, 5.38 and 5.50, respectively. In addition, the organic matter from soils of Hangzhou, Nanning and Changsha was 37.2, 32.3 and 8.1 g/kg, respectively, while the cation exchange capacity was 14.20, 20.64 and 6.90 cmol/kg, respectively. In addition, there was a difference in soil texture between the above three soil samples, while the sand content was 35.50%, 23.79% and 16.74%, respectively, the silt content was 34.00%, 34.29%, and 76.69%, respectively, the clay content was 29.50%, 36.85% and 6.57%, respectively (Table 6).

**Table 6:** Characteristics of orchard soils that were sprayed with glyphosate aqueous solution.

Soil type	Site	pH	CEC (cmol/kg)	Organic Matter (g/kg)	Soil Texture		
					Sand (%)	Silt (%)	Clay (%)
Red soil	Hangzhou, Zhejiang	6.34	14.20	3.72	35.50	34.00	29.50
Clay	Nanning, Guangxi	5.38	20.64	3.23	23.79	34.29	36.85
Red soil	Changsha, Hunan	5.50	6.90	0.81	16.74	76.69	6.57

CEC: Cation exchange capacity.

### 3.4. Measurement of Soil Parameters

Soils collected from both experiment I and II were further characterized as described above by measuring their physicochemical properties such as soil type, pH, cation exchange capacity, organic matter, the content of sand, silt and clay, which were determined based on the standard procedures as described in a number of previous studies [6,27,28,29,30].

### 3.5. Standard Curve of *Glyphosate* and Its Metabolite AMPA

Standard curves of glyphosate and its metabolite AMPA in ddH_2_O and Zhejiang soil extract were drawn in this study based on the linear relation between peak area of HPLC and the injection volume of samples. Glyphosate and its metabolite AMPA solutions at 0.002, 0.01, 0.05, 0.10, 0.50 and 1.00 mg/L were prepared by adding different amount standard sample of glyphosate and its metabolite AMPA into ddH_2_O and Zhejiang soil extract, respectively.

### 3.6. Sample Preparation

Impurities were removed from soil samples that were collected from each site by using a sieve and then about 10.00 g was weighed into a 250 mL plastic centrifuge bottle, 40 mL 0.1 mol/L ammonium hydroxide was added, followed by shaking for 30 min, and centrifuging at 3000 rpm/min for 10 min. After collecting the water layer, the pellet was added with 40 mL 0.1 mol/L ammonium hydroxide, fully shaked for 30 min at 25 °C, centrifuged at 3000 rpm/min for 10 min, and the water layer was merged with the one above, water was added up to 100 mL, then 30 mL were taken and centrifuged at 10,000 r/min for 10 min, 1 mL suspension was added with 1 mL 0.1 mol/L aqueous solution of sodium borate, 2 mL 0.1% FMOC-chloride-acetone solution, and shaken for 2 min. After remaining in a stable state for 20 min, 6 mL ethyl acetate was added, shaked for 1 min and then kept stable for 20 min, before the water phase was taken for HPLC analysis.

### 3.7. HPLC Analysis

Soil samples were reacted with 9-fluorenylmethyl chloroformate and acetone, and the residues of glyphosate and its metabolite AMPA in soil solutions were determined by fluorescence detection using high performance liquid chromatography (HPLC, Waters 2695/2475, Milford, MA, USA), which was carried out as described below. HPLC with an excitation wavelength of 254 nm, emission wavelength of 315 nm, chromatographic column: Phenosphere 5 μ SAX 80A (250 mm × 4.6 mm, 5 µm), column temperature: 50 °C, mobile phase: 0.1 M KH_2_PO_4_ solution: 0.1 M phosphoric acid water solution: water: acetonitrile = 2:12.5:35.5:50, flow rate: 1.5 mL/min, Injection volume: 20 µL.

## 4. Conclusions

Taken together, the data from our study demonstrated that the amount of glyphosate and AMPA residue was reduced with the increase of time after the spraying of glyphosate aqueous solution and soluble powder. Furthermore, there was a marked difference in the degradation dynamics of glyphosate and AMPA between medium loam from Zhejiang and Guangdong Province as well as brown loam from Guizhou Province. In particular, glyphosate soluble powder and its AMPA were well degraded in the medium loam from Guangdong Province, whereby the glyphosate and AMPA residue in this kind of soil was less than 0.10 µg/g at 42 days and 1 day, respectively, after the spraying of glyphosate herbicide. Like soluble powder, a difference in the amount of glyphosate aqueous solution residue was observed between red soil from Hunan and Zhejiang Province, as well as clay from Guangxi Province. Overall, these findings demonstrated that the degradation dynamics of glyphosate aqueous solution and soluble powder as well as AMPA depend on the physicochemical properties of the soils, in particular soil pH, which should be carefully considered in the application of glyphosate herbicide.

## References

[1] Stephen O.D., Stephen B.P. (2008). Glyphosate: A once-in-a-century herbicide: Mini-review. Pest Manag. Sci..

[2] Steinrücken H.C., Amrhein N. (1980). The herbicide glyphosate is a potent inhibitor of 5-enolpyruvyl-shikimic acid-3-phosphate synthase. Biochem. Biophys. Res. Commun..

[3] Schönbrunn E., Eschenburg S., Shuttleworth W.A., Schloss J.V., Amrhein N., Evans J.N., Kabsch W. (2001). Interaction of the herbicide glyphosate with its target enzyme 5-enolpyruvylshikimate 3-phosphate synthase in atomic detail. Proc. Natl. Acad. Sci. USA.

[4] Mink P.J., Mandel J.S., Sceurman B.K., Lundin J.I. (2012). Epidemiologic studies of glyphosate and cancer: A review. Regul. Toxicol. Pharmacol..

[5] Kier L.D., Kirkland D.J. (2013). Review of genotoxicity studies of glyphosate and glyphosate-based formulations. Crit. Rev. Toxicol..

[6] Albers C.N., Banta G.T., Hansen P.E., Jacobsen O.S. (2009). The influence of organic matter on sorption and fate of glyphosate in soil—Comparing different soils and humic substances. Environ. Pollut..

[7] Yamada T., Kremer R.J., de Camargo e Castro P.R., Wood B.W. (2009). Glyphosate interactions with physiology, nutrition, and diseases of plants: Threat to agricultural sustainability?. Eur. J. Agron..

[8] Islas G., Rodriguez J.A., Mendoza-Huizar L.H., Perez-Moreno F., Carrillo E.G. (2014). Determination of glyphosate and aminomethylphosphonic acid in soils by HPLC with pre-column derivatization using 1,2-naphthoquinone-4-sulfonate. J. Liquid Chromatogr. Relat. Technol..

[9] Aparicio V.C., de Geronimo E., Marino D., Primost J., Carriquiriborde P., Costa J.L. (2013). Environmental fate of glyphosate and aminomethylphosphonic acid in surface waters and soil of agricultural basins. Chemosphere.

[10] Zhang C.P., Zhao H., Cai X.M., He H.M., Zhu Y.H., Li Z. (2012). Residue analysis and degradation dynamics of tebuconazole in rice. Agrochemicals.

[11] He H.M., Zhang C.R., Zhu Y.H., Zhang C.P., Ping L.F., Zhao H., Wu M., Tang T., Cai X.M., Li Z. (2014). Residue and degradation of cyantraniliprole and its main metabolite in pepper and soil. Chin. J. Anal. Chem..

[12] Li Y.F., Zhang C., Yin Y.H., Cui F., Cai J.Y., Chen Z.H., Jin Y.H., Robson M.G., Li M., Ren Y.T. (2014). Neurological effects of pesticide use among farmers in China. Int. J. Environ. Res. Public Health.

[13] Lesmes-Fabian C., Binder C.R. (2013). Pesticide flow analysis to assess human exposure in greenhouse flower production in Colombia. Int. J. Environ. Res. Public Health.

[14] Kim J.H., Kim J., Cha E.S., Ko Y., Kim D.H., Lee W.J. (2013). Work-related risk factors by severity for acute pesticide poisoning among male farmers in South Korea. Int. J. Environ. Res. Public Health.

[15] Piel S., Baures E., Thomas O. (2012). Contribution to surface water contamination understanding by pesticides and pharmaceuticals, at a watershed scale. Int. J. Environ. Res. Public Health.

[16] Pasiani J.O., Torres P., Silva J.R., Diniz B.Z., Caldas E.D. (2012). Knowledge, attitudes, practices and biomonitoring of farmers and residents exposed to pesticides in Brazil. Int. J. Environ. Res. Public Health.

[17] Chowdhury M.A.Z., Banik S., Uddin B., Moniruzzaman M., Karim N., Gan S.H. (2012). Organophosphorus and carbamatepesticide residues detected in water samples collected from paddy and vegetable fields of the Savar and DhamraiUpazilas in Bangladesh. Int. J. Environ. Res. Public Health.

[18] Munro I.C. (2000). Safety evaluation and risk assessment of the herbicide Roundup and its active ingredient, glyphosate, for humans. Regul. Toxicol. Pharm..

[19] Catrinck T.C.P.G., Dias A., Aguiar M.C.S., Silverio F.O., Fidencio P.H., Pinho G.P. (2014). A simple and efficient method for derivatization of glyphosate and AMPA using 9-fluorenylmethyl chloroformate and spectrophotometric analysis. J. Braz. Chem. Soc..

[20] Nagatomi Y., Yoshioka T., Yanagisawa M., Uyama A., Mochizuki N. (2013). Simultaneous LC-MS/MS analysis of glyphosate, glufosinate, and their metabolic products in beer, barley tea, and their ingredients. Biosci. Biotechnol. Biochem..

[21] Botero-Coy A.M., Ibanez M., Sancho J.V., Hernandez F. (2013). Improvements in the analytical methodology for the residue determination of the herbicide glyphosate in soils by liquid chromatography coupled to mass spectrometry. J. Chromatogr. A.

[22] Zhang Y.Y., Zhang Y., Qu Q.S., Wang G.X., Wang C.Y. (2013). Determination of glyphosate and aminomethylphosphonic acid in soybean samples by high performance liquid chromatography using a novel fluorescent labeling reagent. Anal. Methods.

[23] Goscinny S., Unterluggauer H., Aldrian J., Hanot V., Masselter S. (2012). Determination of glyphosate and its metabolite AMPA (aminomethylphosphonic acid) in cereals after derivatization by isotope dilution and UPLC-MS/MS. Food Anal. Methods.

[24] He H.M., Zhao H., Zhang C.R., Zhu Y.H., Ping L.F., Wu M., Zhang C.P., Cai X.M., Li Z. (2013). Determination of abamectin residues in grain by ultra-performance liquid chromatography-tandem mass spectrometry. Chin. J. Anal. Chem..

[25] Zhang C.P., Zhao H., Wu M., Zhang C.R., Hu X.Q., Ping L.F., Li Z. (2012). Residue analysis and degradation dynamics of fenoxanil in rice. Acta Agric. Zhejiangensis.

[26] Zhang C.P., Liu X.G., Xu J., Dong F.S., Zheng Y.Q. (2010). Determination of imazethapyr residues in soil using SPE and UPLC-MS/MS. J. Agro-Environ. Sci..

[27] Paramasivam M., Banerjee H. (2012). Degradation dynamics of flubendiamide in different types of soils. Bull. Environ. Contam. Toxicol..

[28] Yu H.Y., Li F.B., Yu W.M., Li Y.T., Yang G.Y., Zhou S.G., Zhang T.B., Gao Y.X., Wan H.F. (2013). Assessment of organochlorine pesticide contamination in relation to soil properties in the Pearl River Delta, China. Sci. Total Environ..

[29] Khandelwal A., Gupta S., Gajbhiye V.T., Varghese E. (2014). Degradation of kresoxim-methyl in soil: Impact of varying moisture, organic matter, soil sterilization, soil type, light and atmospheric CO_2_ level. Chemosphere.

[30] Zhang K.K., Hu D.Y., Zhu H.J., Yang J.C., Song B.A. (2014). Enantioselective degradation of dufulin in four types of soil. J. Agric. Food Chem..

[31] Ghafoor A., Jarvis N.J., Thierfelder T., Stenström J. (2011). Measurements and modeling of pesticide persistence in soil at the catchment scale. Sci. Total Environ..

[32] Borggaard O.K., Gimsing A.L. (2008). Fate of glyphosate in soil and the possibility of leaching to ground and surface waters: A review. Pest Manag. Sci..

[33] Ghafoor A., Moeys J., Stenström J., Tranter G., Jarvis N. (2011). Modeling spatial variation in microbial degradation of pesticides in soil. Environ. Sci. Technol..

[34] Pipke P., Amrhein N. (1988). Degradation of the phosphonate herbicide glyphosate by *Arthrobacter atrocyaneus* ATCC-13752. Appl. Environ. Microbiol..

[35] Kryuchkova Y.V., Burygin G.L., Gogoleva N.E., Gogolev Y.V., Chernyshova M.P., Makarov O.E., Fedorov E.E., Turkovskaya O.V. (2014). Isolation and characterization of a glyphosate-degrading rhizosphere strain, *Enterobacter cloacae* K7. Microbiol. Res..

[36] Fan J.Y., Yang G.X., Zhao H.Y., Shi G.Y., Geng Y.C., Hou T.P., Tao K. (2012). Isolation, identification and characterization of a glyphosate-degrading bacterium, *Bacillus cereus* CB4, from soil. J. Gen. Appl. Microbiol..

